# The Effect of Pulling Out Cochlear Implant Electrodes on Inner Ear Microstructures: A Temporal Bone Study

**DOI:** 10.1155/2011/107176

**Published:** 2011-10-11

**Authors:** Ingo Todt, Rainer O. Seidl, Arne Ernst

**Affiliations:** Department of Otolaryngology, Unfallkrankenhaus Berlin, 12683 Berlin, Germany

## Abstract

The exchange of an cochlear implant or the re-positioning of an electrode have become more frequently required than a decade ago. The consequences of such procedures at a microstructural level within the cochlea are not known. It was the aim of the present study to further investigate the effects of an CI electrode pull-out. Therefore 10 freshly harvested temporal bones (TB) were histologically evaluated after a cochlear implant electrode pull-out of a perimodiolar electrode. In additional 9 TB the intrascalar movements of the CI electrode while being pulled-out were digitally analysed by video- capturing. Histologically, a disruption of the modiolar wall or the spiral osseous lamina were not observed. In one TB, a basilar membrane lifting up was found, but it could not be undoubtedly attributed to the pull-out of the electrode. When analyzing the temporal sequence of the electrode movement during the pull-out, the electrode turned in one case so that the tip elevates the basilar membrane. The pull- out of perimodiolarly placed CI electrodes does not damage the modiolar wall at a microstructural level and should be guided (e.g., forceps) to prevent a 90 o turning of the electrode tip into the direction of the basilar membrane.

## 1. Introduction

Cochlear implant (CI) surgery has become a widespread procedure with clearly increasing numbers around the world to treat deaf and profoundly hearing impaired patients of different age.

Histological studies of human cochleae have evidenced different levels of intracochlear trauma induced by the insertion of different CI electrode arrays [1–3]. These reports led to the development of specific insertion techniques [4] and tools [5] for perimodiolar electrodes to minimize the insertion trauma as found earlier with this kind of electrodes [1, 6]. 

Over time, the technology of histological workup of inserted cochleae was refined so that it became possible to investigate cochlear structures with the positioned array in place [7]. Before this technological advancement, investigations of cochlear structures were limited insofar that the inserted electrodes had to be removed before workup of the cochlear tissues [8–10]. Thus, it was unclear if observed changes were due to the insertion or the pull-out of the electrode array. 

The development of those specific insertion techniques showed a limited insertion trauma, but the basilar membrane and the osseous lamina spiralis are at limited risk [4, 11, 12]. 

It is therefore of particular interest for surgeons to identify risk factors and structures if an electrode pull-out becomes necessary for several clinical reasons, for example, an exchange of the implant (due to failure), an intraoperative pull-out of an already inserted electrode in order to replace it by a back-up device, and so forth. 

The aim of the present study was to investigate possible, intracochlear changes after insertion and extraction of a perimodiolar electrode with state-of the-art traumatic insertion technique and refined electrodes.

## 2. Methods

### 2.1. Temporal Bone Preparation for Histological Evaluation

The temporal bones were freshly harvested and subsequently worked up. At first, a conventional mastoidectomy with a posterior tympanotomy was performed. The CI electrodes (see below) were inserted with the AOS technique after removal of the promontory lip, preparation of the round window membrane, and opening and inferior enlargement of the round window [13]. Subsequently, the array was carefully pulled out (see also below). The stapes were removed, and the labyrinth was perfused with formalin (4%). Then the temporal bones were divided into two groups for further histological analysis.

- In 5 fresh TBs, a methylmetacrylat embedding technique was performed. The TB were thin-sliced (200 ym), polished.
- 5 separate TBs were decalcified by EDTA, fixed by paraffine, thin-sliced (4 ym), and stained by haematoxylin-eosin (HE).

### 2.2. Image Analysis of the Pull-Out Procedure

In a separated number of 9 TBs, the bony roof of the scala vestibuli was removed so that a full overview of the basilar membrane was posssible. After insertion of the electrodes, the pull-out movement of the electrode was digitally frame-captured and analysed via an attached video system.

### 2.3. Electrodes Applied in the Experiments

All TB experiments were done with Nucleus Advance electrodes (Cochlear Corporation, Sydney, Australia).

All temporal bone donors gave their written consent beforehand. The study was approved by the Institutional Review Board.

## 3. Results

### 3.1. Metacrylate Embedding of TB (Series A)

**Figure 1:**
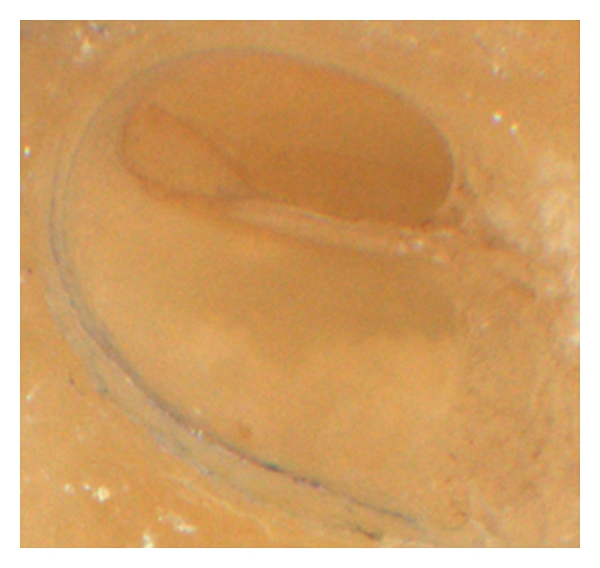
Scalaer overview. No sign of pull-out-related lesion.

**Figure 2:**
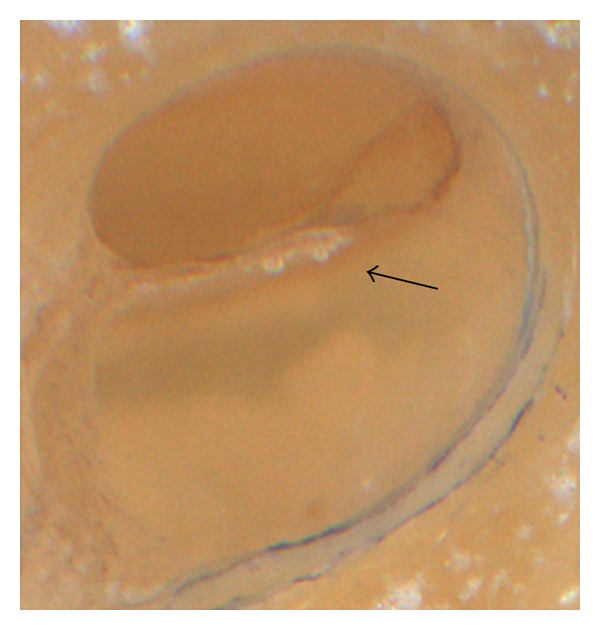
BM lifting. Bubble formation with tissue shearing under the BM as a sign of BM lifting.

The histological analysis of the cochlear microstructures revealed no major changes in all 5 TBs, particularly not at or around the modiolar wall (Figures 1 and 2). In one TBs, the basilar membrane was elevated up at the basal part of the scala tympani (Figure 2). However, neither a disruption of the basilar membrane was found, nor was a breakup of the osseous lamina spiralis. The lateral cochlear wall was also without changes (Figures 1 and 2). If this finding should be classified due to the so-called “intracochlear trauma scale” by Eshraghi et al. [1], one grade 1 trauma was found (Table 1).

### 3.2. EDTA Staining of TB (Series B)

**Figure 3:**
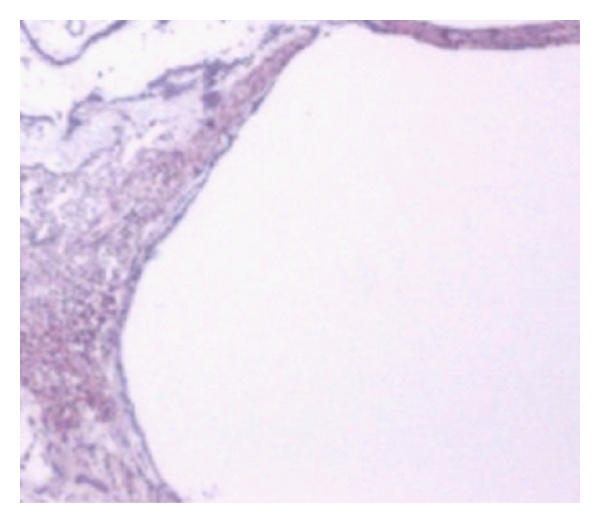
Exemplaric regular modiolar wall.

The histological microanalysis revealed no microtraumatization of any intracochlear structure in any of the 5 TBs in series B (Figure 3) (see also Table 2).

### 3.3. Image Analysis of the Pull-Out Procedure

**Figure 4:**
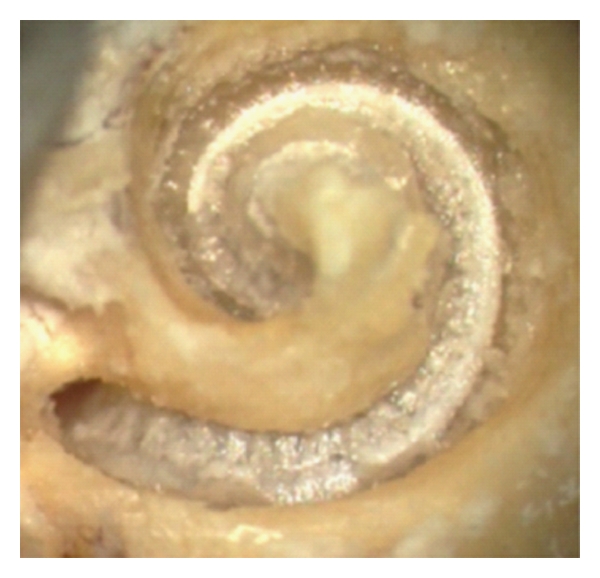
Cochlea with preperated basilar membrane before pull-out. CI array positioned under the BM.

**Figure 5:**
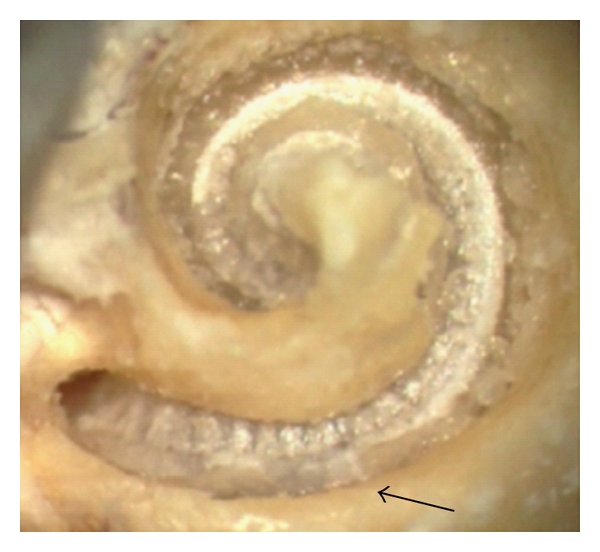
Cochlea with preperated basilar membrane while pulling out. Regional reflex change of the basilar membrane with the array tip underneath as sign of a BM elevation.

The analysis of the pull-out procedure showed in 1 out of 9 TBs that the electrode tip was tilted upwards by a 90° turning of the tip (Figures 4 and 5). When the pull-out was manually controlled by microinstrumentation (forceps) and a gentle movement towards the modiolus, the tip tilting could be prevented.

## 4. Discussion

The impact of cochlear implantation on the fine structure of the inner ear has been a subject of research for some years. The electrode-tissue interface should be investigated on one hand to modify electrode arrays [1–3, 6] and on the other to improve the surgical approach (i.e., the site and extension of the cochleostomy) [14, 15]. This detailed microstructural analysis with the electrode in place was not yet possible in the 80s so that no clear distinction between intracochlear changes due to electrode insertion or extraction could be made. The recent perimodiolar arrays had not yet been the subject of studies since straight arrays dominated the market [16].

Our present study suggests that the modern, perimodiolar electrodes only cause minor trauma to the cochlear microstructure. Apart from a lifted basilar membrane, no serious damage was found even when applying two different techniques as done in series A and B. The modiolus, which is very closely related to the electrode, remained unaffected.

The results of our study also confirm the findings of [4, 11, 12] of a limited trauma with the difference that in our study the electrode was additionally removed. This means that the present electrode arrays can be safely inserted, but even safely removed and/or replaced if required. Some additional features, for example, fluid exchange in between labyrinthine compartments, indirect suction forces have to be considered as well. Our histological findings have some clinical implications. 

A complete pull-out of the electrode might become necessary to improve its position, in case of device failure and/or replacement. Since the modiolar region with its neurons plays a central role in carrying the electrical stimulus to the other regions of the auditory pathway, its proven integrity after a complete electrode pull-out lets us suggest that a limited pullback of a CI electrode array to better approximate the electrode to its neural interface bears no serious risks to the cochlear microstructures [13, 17]. The audiological results after a device replacement support this suggestion [18, 19].

The only minor change after electrode extraction was found at the basilar membrane as related to an upward tilting of the electrode tip. However, we could not finally differentiate between an insertional or an extractional mechanism. The video analysis revealed the tip turning during the extraction of the array (Figures 4 and 5) but as outlined above, a gentle and guided pull-out of the electrode (e.g., with forceps) can prevent a lifting-up of the basilar membrane by a tilted electrode tip.

## 5. Conclusion

Perimodiolar electrode pull-out does not damage the modiolar wall in vitro. While pulling out the cochlear implant electrode, this procedure should be manually guided to prevent damage to the basilar membrane.

## Figures and Tables

**Table 1:** Metacrylat preparation: results after pull out.

TB	basil. memb.	mod.	lat. wall
S66/06	reg	reg	reg
S69/06	reg	reg	reg
S78/06	lift.	reg	reg
S79/06	reg	reg	reg
S80/06	reg	reg	reg

**Table 2:** EDTA preparation: results after pull-out.

TB	bas. mem.	modiolus	lat. wall
S62/06	reg	reg	reg
S27/09	reg	reg	reg
S26/09	reg	reg	reg
S83/08	reg	reg	reg
S17/09	reg	reg	reg
